# Understanding the barriers and facilitators related to never treatment during mass drug administration among mobile and migrant populations in Mali: a qualitative exploratory study

**DOI:** 10.1136/bmjgh-2024-015671

**Published:** 2024-10-09

**Authors:** Moussa Sangare, Abdoul Fatao Diabate, Yaya Ibrahim Coulibaly, Diadje Tanapo, Sekou Oumarou Thera, Housseini Dolo, Ilo Dicko, Oumar Coulibaly, Binta Sall, Fatoumata Traore, Seydou Doumbia, Manisha A Kulkarni, Thomas B Nutman, Alison Krentel

**Affiliations:** 1 International Center of Excellence in Research in Mali, University of Sciences, Techniques, and Technologies of Bamako, Bamako, Mali; 2 Interdisciplinary School of Health Sciences, University of Ottawa, Ottawa, Ontario, Canada; 3 Bruyère Research Institute, Ottawa, Ontario, Canada; 4 Dermatology Hospital of Bamako, Bamako, Mali; 5 School of Epidemiology and Public Heath, University of Ottawa Faculty of Medicine, Ottawa, Ontario, Canada; 6 Laboratory of Parasitic Diseases, National Institute of Allergy and Infectious Diseases, Bethesda, Maryland, USA

**Keywords:** Public Health, Control strategies, Infections, diseases, disorders, injuries, Systematic review

## Abstract

**Introduction:**

Five of the neglected tropical diseases use a strategy of preventative chemotherapy distributed via mass drug administration (MDA) for all eligible people living in endemic areas. To be successful, high coverage must be sustained over multiple rounds. Therefore, it will be difficult to reach elimination as a public health problem using MDA if there remain clusters of people who have never been treated. The study aims to explore the reasons why people with high mobility report being never treated during MDA and to provide evidence to support the development of standardised questions for data collection using qualitative research tools.

**Methods:**

We conducted an exploratory study using qualitative methods among displaced people, nomads/transhumants and economic migrants who self-reported that they had never been treated during MDA in the health districts of Tominian and Kalabancoro in Mali. Data were collected through in-depth individual interviews and focus group discussions. Nvivo V.14 software was used for data management and analysis.

**Results:**

The main reasons reported for never treatment included: geographical mobility, lack of awareness/information, negative rumours, fear of side effects, conflict and insecurity and logistical difficulties faced in reaching these populations. Proposed solutions included involving communities in the MDA, increasing awareness and information campaigns, effectively managing side effects, and designing and implementing flexible and effective interventions.

**Conclusion:**

This study highlights that there are people with high mobility who may never have been treated during any round of MDA. The reasons for never treatment highlight the challenges faced when reaching particular groups during MDA activities/interventions. Suggested remedies will require programmes to implement more flexible and tailored interventions. Customised approaches based on the context are essential to guarantee fair access to preventive chemotherapy. Effective interventions must consider the supply and demand side in crafting interventions. This research adds to the evidence base to understand never treatment, particularly among highly mobile population groups and in schistosomiasis elimination programmes.

**WHAT IS ALREADY KNOWN ON THIS TOPIC**

**WHAT THIS STUDY ADDS**

- This is one of the first papers that describes the reasons why mobile populations may remain untreated during mass drug administration (MDA) for NTD control and elimination.
- Analysed using Levesque’s framework for access to healthcare, the reasons leading to never treatment highlight entry points to reach particular groups during MDA interventions.

**HOW THIS STUDY MIGHT AFFECT RESEARCH, PRACTICE OR POLICY**

- The study highlights the impact of significant geographic mobility of certain populations in Mali on the success of MDA efforts.
- This research provides insight towards the creation of a standardised and successful programmatic response.
- A universal strategy may not be adequate, and customised approaches are needed to address the contextual differences of highly mobile populations.
- The use of Levesque’s framework provides guidance to assess the supply and demand sides of MDA in order to improve access for mobile populations.

## Introduction

 In sub-Saharan Africa, neglected tropical diseases (NTDs) are common, contributing to approximately 40% of the global burden of NTDs, with about 600 million individuals still requiring preventive chemotherapy (PC) treatment.^1^ An essential part of the strategy for some NTDs uses PC administered through mass drug administration (MDA) without individual diagnosis. MDA is a safe and affordable public health intervention, in part due to the provision of donated medicines.^2^ The WHO recommends PC for five NTDs (PC-NTDs): trachoma, lymphatic filariasis (LF), onchocerciasis, schistosomiasis and soil-transmitted helminthiases (STH).^3^

The global fight against NTDs has achieved substantial success, reducing morbidity and mortality, and improving health and well-being for millions of people.^4^ As of 2024, 19 countries in the WHO African region have eliminated at least one NTD.^5^ Trachoma has been eliminated in the Gambia, Ghana, Malawi, Togo, Benin and Mali, while LF has been eliminated in Togo and Malawi.^6^ To date, Togo has eliminated four NTDs: Guinea worm (2011), LF (2017), human African trypanosomiasis (2020) and trachoma (2022).^7^ Mali has had a historically high prevalence of NTDs, yet the national programme has made significant progress using MDA interventions, eliminating trachoma in 2023 and submitting a dossier for LF elimination certification in 2026.^8 9^ MDA for LF and STH ceased in 2019, and Mali is on track to eliminate onchocerciasis, however, schistosomiasis remains a challenge, with all districts considered as endemic.^10 11^

As many countries move towards the endgame of a particular NTD, new strategies are needed to meet the targets outlined in the WHO 2030 NTD Roadmap.^12^ Increasingly, the importance of never treatment has been raised as a potential risk to elimination goals for PC-NTDs.^13^ Never treatment is defined as a self-reported statement of never taking the PC offered during any round of MDA.^14^,^17^ This group of people are termed as ‘never treated’ and are estimated to be more than 25% of the eligible population in some locations.^18^ Untreated individuals risk being reservoirs for parasites, leading to disease re-emergence.^19^ Research has suggested that never treated individuals may be greater risk for infection with LF.^20^,^22^ Recent modelling suggests that increased proportions of never treatment can result in increased MDA rounds and delay elimination for LF.^23^ Similar modelling for STH and schistosomiasis MDA programmes suggests that never treatment impacts the potential for elimination of both parasites.^18 24 25^ As part of the challenges facing the NTD community, never treatment has become a critical aspect that must be addressed elimination goals are to be reached.

Despite Mali’s notable progress in MDA campaigns against PC-NTDs, there is a concern that clusters of never treated people could risk the programmatic gains made in the last decade particularly given the recent geopolitical shifts and history of population mobility within the country. From 2008 to 2022, Mali has seen 380 000 internally displaced people (IDPs), representing an important increase in total IDPs within the country.^26^ Nomads and transhumants move seasonally to find food and water for animals, however, due to an increasingly volatile security situation in some parts of the country and the impacts of climate change, their internal migration has also increased. In the context of this study we have defined nomads or nomadic populations as groups of people who temporarily or permanently move their residence and work activities from one place to another. Nomads encompass various groups such as nomadic hunter-gatherers, pastoralists and peripatetic communities (which are groups moving among settled populations while offering a craft or trade). Pastoralists can be further categorised as (1) transhumants (nomadic groups regularly migrating between two grazing areas along established routes), (2) pastoralists migrating along traditional routes but also exploring different areas annually and (3) semi-pastoralists exhibiting semi-sedentary living and movement patterns.^27^

Mobile populations, including IDPs, nomadic/transhumant herders, refugees and migrants, often have limited access to public health interventions due to their mobility.^28 29^ Studies have shown that MDA and routine healthcare delivery among mobile populations are difficult to achieve and result in missed treatments.^30 31^ It is therefore expected that some people will miss annual MDA treatment and some will never be treated during any MDA round. Never treatment is particularly important for Mali PC-NTD programmes because Mali has stopped MDA for trachoma, STH and LF with the Stop MDA surveys in progress for onchocerciasis. There are concerns that the intersection of highly mobile populations and never treatment could be a risk for re-emergence of some NTDs in Mali. The reasons for missed treatment vary over time but may include ignorance, fear of side effects, cultural beliefs, lack of risk perception, absence, ineligibility such as pregnancy, community drug distributors (CDDs) profile or attitude.^32^,^35^

To address never treatment, special attention for mobile populations is needed. Numerous studies indicate that population movement plays an important role in the spread and control/elimination of NTDs.^36^,^38^ In the context of NTD elimination, understanding why some members of mobile populations are never treated and identifying ways to reach them will be useful in achieving sustainable elimination goals in Mali. This study aims to understand the barriers and facilitators related to never treatment during MDA for one of the PC-NTDs, schistosomiasis, among mobile and migrant populations in Mali; to explore how programmes may broaden their reach. Finally, we aim to make recommendations for an operational response to never treatment issues based on the study outcomes.

## Methods

### Study setting

The study was carried out in the health districts of Tominian and Kalabancoro. These two districts were selected in collaboration with the national NTD programme according to the following criteria: presence of diverse highly mobile population groups, MDA for schistosomiasis has been completed recently and an acceptable security situation that allows the research team to travel.

Tominian’s health district is a rural area in the Segou region, 465 km from the capital city of Bamako. This district is known to have significant transhumance movements, IDPs, nomads and economic migrants between the Bourgou (a geographical area within the health district of Koro), the Djenné health district in Mopti region at the centre of the country, and the bordering districts with Burkina Faso. The main economic activities of people living in the district include farming, cattle breeding and trading. Most people in this region originate from northern and central Mali, and because of insecurity there, many have brought their grazing animals to this region where there is more security and grass.

**Figure 1:**
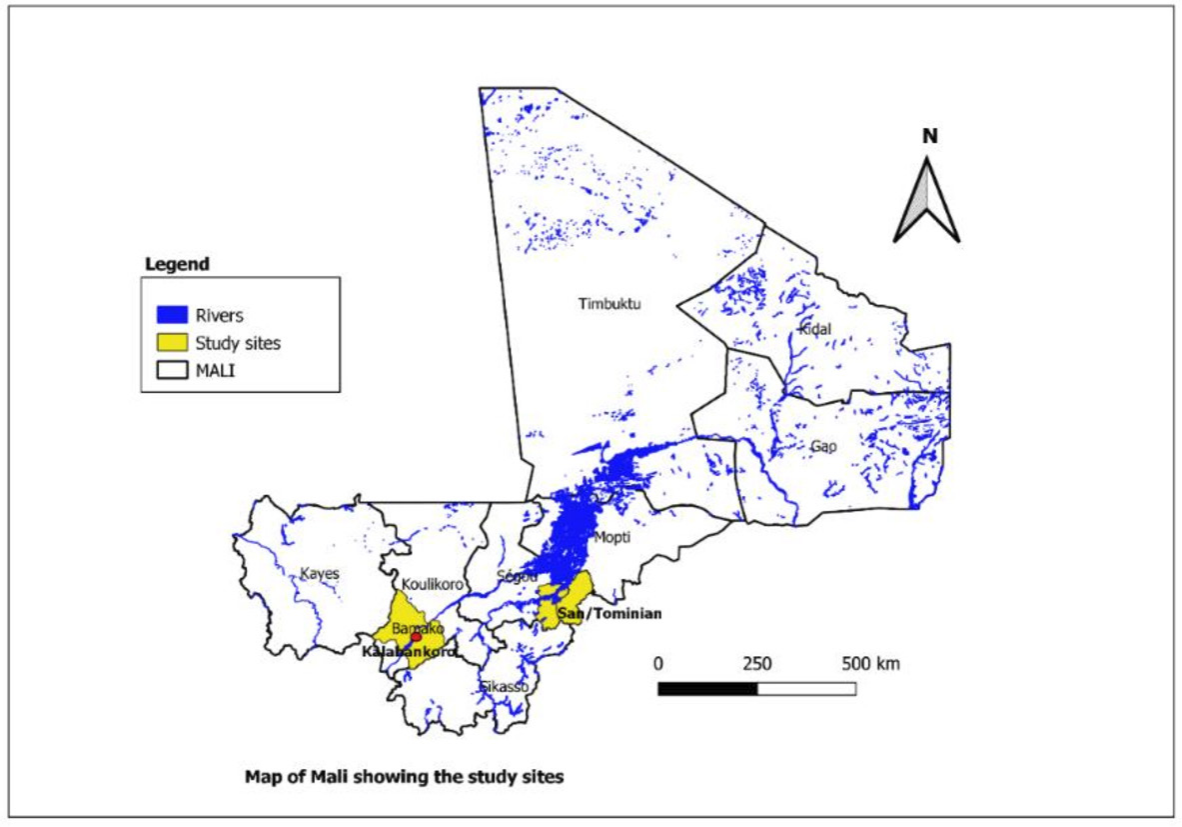
Map of Mali showing the main study health districts. Source: NTDs Research Unit, ICER-Mali, 2024.

Kalabancoro district is a suburban environment, where the population is characterised by economic migrants and informal gold and sand miners, and IDPs from the northern part of Mali. With a population of 166 722 inhabitants, the city, which is the third most populous in the country, is situated on the southern bank of the Niger River. The commune has been included in Bamako’s suburbs, which is the capital of Mali (figure 1: map of Mali with study sites).

### MDA history and coverage for both districts

MDA interventions started in Mali with schistosomiasis control in 1979, which became a national programme in 1982 (including STH). In 1986, ivermectin was introduced for onchocerciasis in endemic districts; followed by MDA for trachoma (1998) and for LF (2004). In 2007, MDA activities were integrated for all five NTDs as a pilot implementation in three regions (Kayes, Koulikoro and Sikasso) which included 24 districts. Over the years, the programme expanded gradually, achieving nationwide coverage for the five PC-NTDs by 2009. Coverage rates range from 76% to 100%, and have remained consistently high since 2009.^8^ In some health subdistricts, the number of people treated was even higher than expected, due to unforeseen population movements and the fact that the census is not regularly updated before each treatment. Currently, in both study districts, only praziquantel distribution for schistosomiasis is ongoing. Since 2022, MDA for schistosomiasis has been carried out by subdistrict, with distribution taking place both in the community and at schools.

In Tominian, the last MDA in 2023 distributed praziquantel. Originally the programme targeted 89 230 children for treatment, and managed to treat, 97 608, approximately 9% more. This excess is likely due to the inclusion of IDP populations and migration from other areas. In Kalabancoro, praziquantel was distributed for schistosomiasis in 2023. They achieved 97.46% therapeutic coverage, treating 56 886 out of a planned 58 367 children according to the National Schistosomiasis Control Programme. This study followed the last round of MDA for schistosomiasis in both areas.

### Study design and data collection

This study employed an exploratory design using qualitative research methods, specifically in-depth interviews (IDIs) and focus group discussions (FGDs). A purposive sampling frame was used to identify participants among the different mobile groups.^39^ Participants were recruited across mobility, gender and age ranges (18 years and above). Initially, health records and MDA data identified areas with low treatment coverage. To identify never treated individuals to participate in the IDIs, we met with community leaders and CDDs to explain the study and the concept of ‘never treatment’. Collaborations with local community leaders, and health workers facilitated access to potential informants. Direct outreach and community meetings were employed to explain the study’s goals and ensure confidentiality. They directed us to individuals uninterested in MDA and newcomers like IDPs and nomads. The team informally approached potential participants and gauged their interest in participating. In compliance with the principles of research ethics, we decided not to include those who did not volunteer to take part in our study. At this level, we had planned 24 IDIs with community members (never treated), that is, 12 per district. IDIs were also planned with key informants including healthcare professionals including CDD living in both districts. The planned sampling frame for these IDIs included four CDDs and four healthcare professionals.

A total of eight FGDs were planned with community members who identified as never treated and who lived in the study districts. IDI participants were identified according to population subgroups of interest and invited to participate in the IDIs on the basis of their characteristics of interest, their level of involvement in the MDA process and their availability and willingness to participate in this research. For these FGDs, males and females were split into different groups. Every FGD had a target of between six and eight participants and was led by a note-taker and an interviewer using a structured topic guide. IDIs and FGDs were audio-recorded and had an average duration of 30–45 min. The topic guide ensured that the intended field of inquiry was covered and facilitated the comparison of information gathered from respondents. The development of the topic guide was guided by known themes related to never treatment and healthcare access, and incorporated data collection sheets and seasonal calendars. The guide was iteratively refined throughout the IDIs and FGDs to better address emerging themes and insights. Specific questions were designed to extract details regarding the unique attributes of every MDA, such as target demographics, and implementation methods. We also showed medicines and empty medicine boxes to participants as a memory aid. The FGDs incorporated seasonal calendars, which are a participatory tool that seeks to understand the broader community experiences regarding their movement and activities throughout different seasons.^40^ A template was designed which allowed participants to outline their different activities throughout the year (seasons, movements, harvest, among others). All data collection materials (FGDs and IDIs guides, and seasonal calendar) were tested in Bamako prior to fieldwork.

All FGDs and IDIs were conducted in either Bambara or French at the preferred time and location of the participant(s). Interviews were conducted until data saturation was reached. Individuals conducting the IDIs and FGDs had prior experience and received additional training prior to the study’s commencement to ensure a thorough understanding of the research objectives and methodologies.

### Data management and analysis

Recorded IDIs and FGDs were transcribed and translated into French. To ensure an accurate translation, the investigators exchanged their files and listened again, comparing what was written and what was said in the audio file. If necessary, a third person was called in to reconcile any discrepancies. We did not return Transcripts to participants for review or correction to preserve the integrity of the original data as collected.

Analysis was conducted using thematic analysis,^41^ which is a systematic approach that allowed the research team to identify and interpret recurring themes and patterns in the data. The analysis team co-coded a selected number of transcripts to develop a pre-established codebook. Once agreed on by the coders, this pre-codebook was used to initiate coding using Nvivo V.14. This process was iterative and inductive, that is, each time a member of the coding team found a new theme, he or she communicated with the others to take it into account. At the end of coding, the Nvivo V.14 files were combined to perform the final analysis. The transcripts were analysed using a process of open and axial coding.^42^ Initial open coding involved breaking down the data into smaller segments, and these segments were assigned codes that captured the essence of the content. Axial coding followed to establish connections between codes and to develop broader categories.

Nvivo V.14 software facilitated coding and data management. Coding and analysis were iterative, with constant comparison and refinement of categories. Two team members collaborated to analyse data, ensuring coding process reliability and validity. Through co-coding, team members individually analysed qualitative data using the codebook with established or emergent codes. Subsequently, they compared their findings, resolving any discrepancies to reach consensus. Seasonal calendar data underwent a thorough examination to identify patterns and trends in participant experiences and activities throughout the year. To analyse seasonal calendar data, we used various methods.^43^ These included organising data on weather, illness, labour activities, food scarcity, education, human mobility and other NTD programme activities. Additionally, calendars drawn up by different population groups were compared explore differences in responsibilities and perceptions. The seasonal calendars help to understand how seasonal changes affect population mobility, NTD programme activities and other community indicators.

### Community engagement strategies conducted prior to implementation

Before beginning the fieldwork, the research team identified relevant stakeholders including partnering NGOs in NTDs, local community leaders and NTD control/elimination programme managers. We presented the protocol to the NTD programme managers and asked for their agreement to use the study data. Prior to travelling to the study sites, we contacted the physicians, healthcare providers and administrators of relevant community health centres. The research team also met with village leaders and CDDs at the community level to introduce the study and gain permission to carry out the activities. We also had meetings to discuss the study’s objectives with community leaders at the gold-panning sites. This process was carried out over the course of the study. To carry out the IDI and FGD, we put in place a very good team of interviewers, made up of male and female public health doctors with extensive experience in the social sciences. The team was supervised by senior researchers from the University of Science, Technology and Technologies of Bamako in Mali, the University of Ottawa in Canada and the National Institutes of Health in the USA. While conducting this work, we acknowledged the significance of reflexivity, recognising our position as the researchers and any potential biases that might affect the results of the study (see the reflexivity statement in online supplemental file 1).

###  Patient and public involvement

The research’s design, methodology, reporting and dissemination plans did not involve input from participants or the general public. However, we would like to thank all the participants for their willingness and cooperation in taking part in this study. We would also like to thank all the administrative and local leaders for their support and consent to conduct this study.

## Results

A total of 31 IDIs and 10 FGDs (with 4–5 individuals in each FGD) were included in the final analysis. In Kalabancoro health district, the 6 FGDs were conducted with 3 groups of women and 3 groups of men, the 12 IDIs with CM were conducted with 6 women and 6 men, the 5 IDIs with health professionals were conducted with 1 woman and 4 men. In Tominian health district, the 4 FGDs were conducted with 2 groups of women and 2 groups of men, the 12 IDIs of CM were conducted with 6 women and 6 men and the 2 IDIs with health professionals were conducted with men.

### Participants’ characteristics

**Figure 2:**
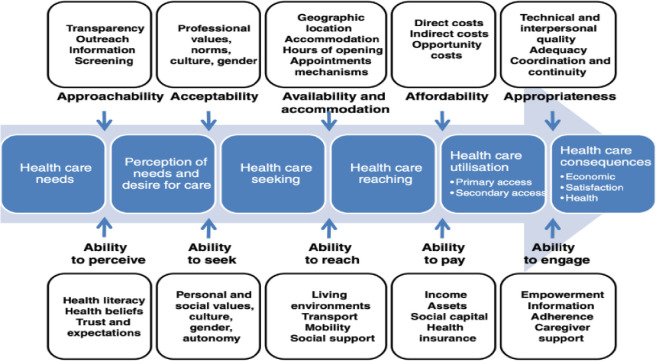
Levesque’s conceptual framework of access to healthcare (Levesque *et al*^44^).

The demographic characteristics of the participants in this study are presented in table 1. Overall, there were more male than female participants for both FGD and IDI. Results are reported according to the Levesque framework^44^ which outlines the supply and demand sides for healthcare access. Levesque and colleagues conceptualised access to healthcare in five key dimensions: (1) approachability; (2) acceptability; (3) availability and accommodation; (4) affordability; and (5) appropriateness. Additionally, the Levesque model highlights five corresponding abilities of the patients or population: (1) ability to perceive; (2) ability to seek; (3) ability to reach; (4) ability to pay; and (5) ability to engage^44^ (figure 2). The research findings are mapped along the parameters of this framework to identify barriers and facilitators relating to never treatment during MDA among mobile and migrant populations in Mali (table 2).

**Table 1:** Characteristics of the overall study population

Type of data collection	Health district	Health subdistrict	Gender		Total
			Female	Male	
FGD	Kalaban-coro	Baguinéda	5	6	11
		Tourela	8	10	18
	Tominian	Fangasso	0	4	4
		San	10	5	15
IDI	Kalaban-coro	Dialakorobougou	0	2	2
		Kalabancoro central	3	3	6
		Kassela	0	2	2
		Tourela	4	3	7
	Tominian	Fangasso	0	1	1
		Monikoro	0	1	1
		Monisso	1	1	2
		San	5	5	10
**Average age of participants by health district**					
	**Health district**	**Minimum**	**Maximum**	**Mediane**	**Q1–Q3**
FGD	Kalaban-coro	24	53	40	28–46
	Tominian	24	44	34	27–41
IDI	Kalaban-coro	19	57	33	27.5–45
	Tominian	17	76	34.5	25.5–49.25
**Study sites**	**FGD**	**IDI (CM)**	**IDI (HP)**	**Seasonal calendar**	**Total**
Kalaban-coro					
Female	3	6	1	2	12
Male	3	6	4	2	15
Tominian					
Female	2	6	2	2	12
Male	2	6	0	3	11

CM, community member; FGD, focus group discussion; HP, health professional; IDI, in-depth interview.

**Table 2:** Barriers and facilitators of access to mass drug administration identified by participants in in-depth interviews and focus group discussions using Levesque *et al*’s framework

Supply-side dimension of accessibility	Themes and their respective barriers (B) and facilitators (F)	Corresponding demand-side dimension of ability to access	Themes and their respective barriers (B) and facilitators (F)
Approachability	Lack of communication channels and low education levels in rural and mobile populations (B) Educational campaigns (F)	Ability to perceive	Beliefs, cultural barriers and misconceptions (B) Lack of communication channels and low education levels in rural and mobile populations (B)
Acceptability	Lack of appropriate training and knowledge of CDD (B) Community health workers’ role (F)	Ability to seek	Lack of appropriate training and knowledge of CDD (B) Discrimination and stigmatisation (B)
Availability and accommodation	Geographic mobility (B) Seasonal observations on population shifts (B) Role of security in the movement (B) Accessible and flexible services (F)	Ability to reach	Geographic mobility (B) Seasonal observations on population shifts (B) Role of security in the movement (B)
Affordability	Economic constraints (B)	Ability to pay	Economic constraints (B)
Appropriateness	Culturally tailored interventions (F)	Ability to engage	Culturally tailored interventions (F)

CDD, community drug distributor.

Furthermore, to ensure a comprehensive reporting on qualitative research methods in the study, the manuscript benefited from the use of the Consolidated criteria for Reporting Qualitative research: a 32-item checklist for interviews and focus groups.^45^ A checklist of items has been included for this paper (online supplemental file 2).

### Approachability and the ability to perceive

Approachability and the ability to perceive healthcare services are crucial supply-side components of the Levesque framework. These elements emphasise the importance of clear communication and the dissemination of information to ensure that individuals are aware of available health services and its benefits. While implementing MDA campaigns in areas where there are high numbers of mobile populations, it is essential to efficiently disseminate information about the dates, times, locations and benefits related to MDA so as to overcome barriers such as misconceptions and language barriers. The study highlighted important barriers and facilitators with respect to approachability and the ability to perceive dimensions such as educational campaigns, lack of communication channels and low education levels in rural and mobile populations, beliefs, cultural barriers, and misconceptions.

### Educational campaigns

Educational campaigns designed to dispel myths, clarify misconceptions and inform migrants about the benefits of MDA can have a positive impact on improving coverage. Results from this research demonstrated that visual aids, such as posters and videos, were particularly helpful in conveying information despite language barriers. In one IDI, one adult male sand miner in Kalabancoro said:

When distributing medicines, advance notice about MDA dates and locations, along with their benefits, should be provided through radio or television. Without information, encountering CDD without advance information becomes difficult to accept. (II_HA_NT_ES_KAL)

### Lack of communication channels and low education levels in rural and mobile populations

The study revealed a poor standard of living and low level of education for people living in endemic rural areas including the mobile populations. These factors are overlooked in planning MDA for Mali’s rural and mobile populations. As a result, there may not be sufficient communication avenues to reach these individuals.

During an FGD, a man stated:

‘I also think there is a lack of communication. Most people here rarely listen to radios, so we need town criers to carry the message’ (FGD_FJ_NT_ES_KAL).

So, if that is the way it is going to be, we need to make sure we share information with all these internally displaced people to raise their awareness. These internally displaced people often do not receive enough information about the campaigns (II_AS_HJ_KAS_KAL).

### Beliefs, cultural barriers and misconceptions

Cultural conceptions can affect how people see medical treatments such as MDA. Some cultures have traditional beliefs that oppose taking biomedical or Western medication, or they may harbour misgivings about modern medicine. Without sufficient communication and outreach, community members may not perceive the benefit of MDA to address the health needs in their household, or their community and preferences for traditional medicines may persist. One young male community member stated:

I don’t like tablets and haven’t taken one since childhood. I prefer traditional concoctions to tablets and needles. The last time I was treated with tablets and injections was a very long time ago. I just don’t like it (II_HJ_NT_SIA_TOM).

Another female participant said:

Imagine when you go to the hospital even lacking ten (10) CFA francs they deprive you of your medicine. So, in this context, if someone comes to give you these medicines for free you can be afraid and create rumors about these medicines. Some people say that these drugs even make you sterile (II_FJ_NT_MED_TOM).

Participants from mobile populations interviewed for this research expressed their preference for traditional healers and cultural practices over modern medical treatment due to deeply ingrained beliefs. Lower costs and the perceived ability to treat various diseases further drive their reliance on traditional medicine.

A young male from Tominian said,

Even when I left Abidjan to come here, I didn’t accept to be vaccinated, because I don’t tolerate this kind of treatment. When I go to the hospital for a consultation, I do not tolerate the smell of medicine circulating there, so if the consultation is going to take a long time, I give up. I prefer traditional medicine (II_HJ_NT_SIA_TOM).

### Acceptability and the ability to seek healthcare

Acceptability and the ability to seek healthcare are the second dimension of access to healthcare in the Levesque framework, referring to cultural and social factors that impact health services such as MDA. The Levesque framework emphasises the importance of acceptability and the ability to seek healthcare in ensuring effective health interventions, such as MDA. Stigma and discrimination, the lack of training and knowledge of CDDs, and the role of the community health worker were identified as key factors with respect to these dimensions.

## Lack of appropriate training and knowledge of CDDs

Research participants expressed their reluctance to participate in MDA. If the CDD is not able to convincingly explain the rationale for taking the treatment, then individuals may not see value in MDA participation and may refuse the tablets.

One of the community members said:

‘Well, we don’t really understand what they’re doing, they just say take it, it’s good for you, but we’re not sick so I prefer to hold back’ (II_HJ_NT_SIA_KAL).

An adult man said,

Normally when drugs are dispensed, information should be given in advance about when and where they are dispensed, as well as their usefulness. Distributors need to make more effort to tell us about the drugs. If this isn't done and you come across the distributors without having received enough information beforehand, it’s hard to accept in this case. But if you inform them in advance, they will share the information and they will be more than happy to participate and take the drug (II_HA_NT_ES_KAL).

### Discrimination and stigmatisation

Mobile populations, particularly migrants and internally displaced individuals can face discrimination and stigmatisation from the local population and/or healthcare providers. This can discourage them from seeking medical attention due to fears of mistreatment or negative social interactions, illustrated by a participant in an FGD:

Sometimes, the distributors do not consider us unless they are done with serving all the local people in the village. Also, some people put themselves aside, they do not want to participate in such activities because they think they are foreigners here and that they are not concerned (II_AS_HJ_FAN_TOM).

### Community health workers’ role

Community health workers emerged as key facilitators of MDA acceptance. Participants highlighted the importance of trusted individuals within their communities who could explain the benefits of MDA, address concerns and provide information in local languages. During an FGD, a young man said,

‘I will never agree to take these medicines unless they go through our village distributors that we know’ (FGD_HJ_NT_TOU_KAL).

Some people are reluctant to take medicines unless they know the distributor. It is important that the distributor is trusted by the villagers, and that he or she is one of them.

Another young man in Tominian stated:

‘There is also the problem of choosing distributors, that is, you have to use well-known agents, known by the villagers, for reasons of trust’ (II_AS_HJ_MON_TOM).

### Availability, accommodation and the ability to reach

The third dimension of access to healthcare is the availability and accommodation of services. Services are considered available and accommodating if they are physically accessible, timely and have adequate capacity to meet demand. Geographic mobility and insecurity issues were identified as barriers by the participants while observation of seasonal trends and accessible and flexible services were identified as facilitators.

### Geographic mobility/population movements according to seasons

Geographical mobility influences the implementation of MDA within these populations as they move and miss community-based interventions. Population mobility varies according to seasons and activities. This is illustrated during an IDI with a sand miner who said:

There are more people moving during the rainy season because at that time the river is full, but as soon as the rains stop and the river starts getting empty, people return to their original community location. The work here during the dry season doesn't bring in enough, it’s during the rainy season that there is a lot of work and movements (IDI_AS_NT_ES_KAL).

There’s a lot of movement here. During the dry season, a lot of women travel with their children to Bamako, Ségou, and so on. They go there to work and come back to the village during the rainy season to carry out their regular activities. There are also small movements during the harvest. When the rice fields in a particular village are harvested, there’s an influx of women. Some of these women earn enough money in the countryside. Even now we have children who are in Burkina Faso, they go back and forth (II_AS_HJ_MON_TOM).

However, in the case of traditional gold miners, movement was also reported but in the opposite direction. As noted in one FGD, a young man stated:

‘Yes, it’s during the dry season that the farmers come here before going back when the rainy season is about to start (FGD_HJ_NT_TOU_KAL).

For groups who are constantly moving, conventional communication techniques such as posters and flyers might not be as successful, and the usual MDA procedures may not be effective as illustrated by health personnel in Tominian:

In July and August, most residents are on-site carrying out their activities. The problem is that they are no longer at home during the door-to-door campaign. The campaign is organized in July-August, this is the time for fieldwork. They usually don’t come home until evening. There is no campaigning at night (II_AS_HJ_MON_TOM).

### Role of security in mobility (especially in the case of IDPs)

MDA programmes in Mali face obstacles in treating NTDs, especially among IDPs, due to security and transportation issues. Displacement from insecure regions has caused urban overpopulation, which has resulted in a lack of medications as these additional populations were not accounted for in initial MDA planning. Moreover, armed bandits hinder access to some regions, impeding the delivery of treatments and supplies.

A female IDP said:

‘We moved here because of growing insecurity in central Mali’ (IC_FJ_LAF_TOM).

A health worker said in response to a question about low coverage:

‘For me, this low coverage is due to the numerous movements of these people because of the insecurity’ (II_AS_HJ_FAN_TOM).

During an FGD, a young man was asked about the difficulties they currently face in terms of public health needs, said:

As for diarrhea, it’s all the same with the lack of hygiene. Malnutrition, onchocerciasis, and the other diseases you mentioned are the result of poverty. Many people here have become poor because of insecurity. Furthermore, the health workers are afraid to come to us for treatment because of the insecurity. So, we don’t benefit from the drug distributed during your campaigns*.* (II_AS_HJ_MON_TOM)

### Seasonal observations on population shifts: insights from the seasonal calendar

The data from the seasonal calendars provided some explanations and reasons for the population movements studied in the two health districts. The flow of young people to Kalabancoro is more important after the harvest period (October to December). They usually return to their respective villages around the beginning of the rainy season, that is, May to June. People can still move at any time of the year in this district because of its proximity to Bamako and the availability of several means of transportation including buses (online supplemental figure 1).

In the Tominian area, there are IDPs, either Fulani nomads/transhumants or farmers who have fled their villages or grazing areas due to insecurity in northern and central Mali. In addition, there are people seeking to make money because there are no jobs in their communities of origin. Many have settled down and are starting to integrate with the native population. As a result, they turn to farming in the villages they settle in during the rainy season to feed themselves. Because of insecurity, activity characterised as nomadism/transhumance is lessening in Tominian and San health districts (online supplemental figure 2).

### Accessible and flexible services

Providing accessible MDA services which take the mobility of the population into account was expressed by participants as a facilitator for participation. Setting up mobile clinics or temporary treatment points at locations frequently visited by migrants may increase the likelihood of participation. Increasing the number of distributors in these areas would also help to increase therapeutic coverage as illustrated by this quote by a community health centre director:

I think that if we increase the number of distributor teams and the number of annual treatment rounds, that could be a solution for increasing the number of treated people*.* (II_AS_HJ_FAN_TOM)

Another man (Kalabancoro) said,

‘In the beginning, the health workers who came here had enough means to meet our needs, they came with a lot of medicines. Now they can continue to do so, and take advantage of the opportunity to give us medicines for MDA’ (FGD_FA_NT_BAN_KAL).

### Affordability and the ability to pay

In Levesque *et al*’s framework, affordability and the ability to pay is the fourth dimension affecting access to care. In this dimension, economic constraints were cited as the main factor leading to disparities in healthcare access and outcomes among different population groups. These financial barriers can exacerbate inequalities.

### Economic constraints

MDA interventions in Mali are funded by the Malian government and partners such as the WHO and bilaterial donors and use donated medicines. As a result, the treatments are free for people living in endemic communities. However, individuals still perceive opportunity costs in terms of time and effort spent waiting or travelling to distribution points, which can interfere with work and other essential activities. Economic constraints create scarcity of resources (time, money, energy) and impact decision-making. Many participants expressed a desire for treatment but delayed taking action due to extreme poverty and the need to prioritise their basic needs.

People living in distress are most concerned about their basic needs (shelter, food, security). As a result, preventive health services for NTDs may not be perceived as a priority area. This is illustrated by a young male participant, who said,

Life is really hard here, what we need now is food. We didn’t harvest this year, we came here without harvesting. Our biggest worry is food, if we have enough to eat, the rest will come too, InshaAllah. To be in good health, you need to have enough to eat, and vice versa (II_HJ_NT_MED_TOM).

Another man said*:*

I was in Cameroon looking for a better life. I spent a lot of time there, but unfortunately, I didn’t have enough money, so I decided to come back to Mali. Life is very hard here in our village, so maybe I'll go back to Cameroon or another country (II_HJ_NT_TOU_KAL).

### Appropriateness and the ability to engage

Culturally appropriate community interventions are a main facilitator in the Levesque framework’s ‘appropriateness and ability to engage’ feature. Culturally tailored interventions consider the cultural norms, beliefs, and values of the target population, which increases trust, relevance and accessibility. It can also improve communication, enhancing access to health and address specific health needs or risk factors that may face some particular communities. These types of interventions often involve local leaders, giving them ownership in their own health improvement which can increase community and people’s ability to engage in health programmes.

### Culturally tailored interventions

Research results showed that culturally sensitive MDA campaigns, especially for mobile and migrant populations, could benefit greatly from such approaches. Engaging community leaders is crucial for increasing participation and improving health outcomes. Cultural competence is thus vital in ensuring that public health initiatives are both relevant and effective, as illustrated by this young man during a one-to-one interview:

It’s essential to get the local population involved. Anecdotally, during vaccinations, we've noticed that before people agree to be vaccinated, they take the advice of certain community leaders, and these leaders advise them to take the drug. The intervention and involvement of community leaders is essential to the smooth running of campaigns (II_AS_HJ_MON_TOM)

## Discussion

This research aimed to understand the reasons why people with high mobility were never treated over multiple rounds of community-based MDA for schistosomiasis elimination in two districts in Mali. Few studies have explored why people are never treated over multiple years of MDA of PC for NTDs. Much of the published research on MDA coverage and compliance tends to explore the various factors associated with people not receiving or not taking treatment during the last cycle of MDA (eg, absence during MDA, fear of side effects, perception of not being at risk),^14 46 47^ rather than never treatment over multiple rounds. Consequently, there is a lack of understanding about the contributing factors associated with never treatment and how they may change over time. Some of the known factors associated with never treatment in the literature include lack of information, fear of side effects and lack of risk perception.^14 16 35 48^ However, the current published research on never treatment does not explore this concept within mobile populations. This research explored how subgroups who exhibit unique mobility patterns (eg, nomads, IDPs, sand miners and traditional gold miners) experience complications during the implementation of MDA which can contribute to never treatment. Our research showed that economic migration, nomadism, and seasonal or daily travel were identified as critical factors that intersect with commonly known factors for never treatment, such as lack of information, fear of side effects and low-risk perception.^49^

Seasonal and economic variations in travel patterns pose obstacles to both the supply side and demand side of MDA. Study results demonstrated how these characteristics are dynamic, implying that conventional MDA approaches may not adequately address the demands of these mobile populations and sufficiently provide access to the healthcare delivered during MDA. Given these dynamics and the nature of mobile people, how can health programmes adapt? The Levesque framework on healthcare access provided a guide to categorise the research findings for interpretation and action. The findings imply that it is critical to consider the varied and intricate mobility patterns that define different populations to enhance MDA coverage (demand side) and offer mobile populations high-quality healthcare (supply side) that is adapted to their unique situation. This strategy can lead to a more effective and equitable MDA campaigns, guaranteeing that even the most mobile and historically marginalised people obtain PC during MDA for NTDs, and in this research, schistosimasis.

### Movements dynamic: mobile populations and geographical shifts

The findings highlight Mali’s significant population mobility, identifying obstacles that may hinder MDA success and explaining untreated cases. Tailoring MDA strategies to address subgroup barriers is essential for improved healthcare access, emphasising the need to adapt public health approaches for Mali’s increasingly dynamic population. Similar findings were reported by Vegvari *et al* in 2019 in a study exploring how how human movement complicates the elimination of STH in areas where disparities in MDA coverage exist.^50^ Moreover, population mobility and geographical shifts have implications for public health planners for MDA implementation in Mali. Infected individuals moving between regions may introduce or re-emerge infectious diseases.^51^ Mobile populations are difficult to track by an already under-resourced health system, hindering preventive and curative health efforts. Understanding and even anticipating population movement patterns can help to identify and target high-risk areas for NTDs. Thus, MDA strategies in Mali should adapt to temporal and spatial population dynamics, optimising intervention timing and locations (figure 2). This supply-side intervention has the potential to improve all elements of the demand side of the Levesque framework, the ability to seek, to perceive, to reach, to engage and to pay (in terms of opportunity costs).

### Overlap of multiple groups in movement

**Figure 3:**
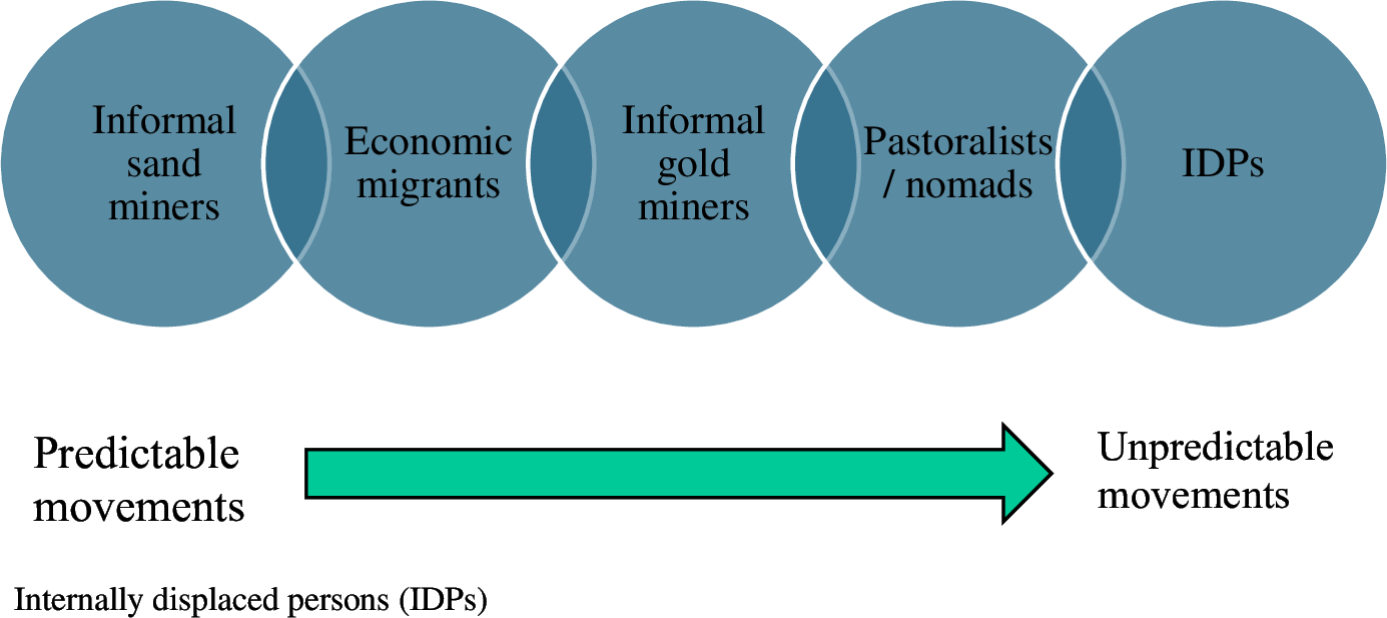
Navigating population dynamics: challenges and innovations in mass drug administration for diverse and mobile communities.

Our study population includes an overlap of multiple groups in movement simultaneously. Some of these movements are predictable, such as those related to seasonal changes, while others, like IDPs, are unpredictable (figure 3). This diversity of population movements has significant implications for the implementation of an effective and equitable MDA. MDA works most effectively in stable communities where people are familiar with one another and tend to stay in the same location for most of the year. It is well-recognised that delivering even basic routine healthcare to mobile populations can be particularly challenging.^52 53^ Consequently, it is reasonable to anticipate that some individuals may be considered as never treated across multiple MDA campaigns, given the dynamic and transient nature of the populations under study in Mali. This emphasises the need for innovative strategies and targeted approaches to reach and serve these diverse and often underserved groups effectively.^54^ This has the potential to improve both the demand and supply sides of the Levesque framework. On the demand side, addressing social and gender norms, geographic barriers and raising awareness, and tailoring services to the living conditions of mobile populations can increase their ability to seek and access healthcare. On the supply side, ensuring service availability and accommodation through mobile teams and more flexible delivery models can better align with the diverse needs of these populations. This holistic approach improves MDA access for mobile groups.

### Security, conflicts issues and access to healthcare

Insecurity and conflict significantly hinder treatment access to healthcare, including MDA. When people flee for safety, they often miss essential treatments. Host districts also face drug shortages, isolation and distrust, further contributing to treatment gaps. Conflict zones frequently report interruptions in MDA introducing potential gaps contributing to NTD elimination goals.^55^ Similar patterns are seen in immunisation programmes where children miss multiple doses or remain zero-dose in conflict areas.^56 57^ Our research findings align with Peprah *et al* who reported non-acceptance of oral cholera vaccination campaigns among IDPs in Juba, South-Sudan during a humanitarian crisis.^58^ Similarly the research in Mali showed that geographic mobility, conflict and insecurity, economic migration, fear of side effects, lack of information/communication, language and cultural barriers and lack of trust in healthcare workers were described as reasons for non-participation in the MDA. Future strategies in Mali where mobile populations are present should consider this phenomenon. Strategies should extend beyond physical security, and relate to the realities experiences by the different kinds of mobile populations.^59 60^ Additionally, the timing of distribution needs careful consideration. In conflict areas, aligning MDA and vaccination campaigns with periods of relative stability can improve participation and coverage.^61^ Tackling these challenges requires a multi-faceted strategy including cross-sectoral collaboration, data sharing, mobile health technologies, flexible healthcare delivery models, CDD teams to target specific groups and community engagement among others. Programmes should consider the complexity of security issues while prioritising population well-being.^59 60 62 63^ NTD programmes need to work closely with mobile populations, local leaders and army as well as populations in insecure and conflict zones to overcome these obstacles. They can also work with organisations with experience working in conflict and insecurity zones. This study emphasises the need for innovative, context-specific solutions that can adapt to the challenges posed by conflict and insecurity, ensuring no one is left behind in MDA delivery. This approach will improve access according to the supply side of the Levesque framework, in particular approachability, availability and accommodation. In addition, by intervening at the supply side, measures might also enhance the demand side of the Levesque framework, specifically the search, the ability to reach (eg, mobility), ability to seek (eg, autonomy) and ability to engage (eg, information and adherence) as mobile populations will have better access to MDA.

### Cultural barriers, beliefs and socioeconomic factors influencing MDA participation

The study reveals that cultural beliefs, misconceptions and rumours impact mobile populations’ MDA participation leaving some people untreated. Our research showed that the fear of drug side effects deterred many people from taking treatment. This has been shown across the schistosomiasis literature on coverage and compliance.^64 65^ Research participants also reported that they do not feel the need to take the treatment during MDA as the risk of infection is not perceived. They do not feel sick or see the illness in their community. This is also a research finding on community uptake of schistosomiasis MDA in other sites.^66^

Mobile populations, particularly for IDPs, face difficulty meeting their basic needs, as this study has shown. This reduces the importance of preventive treatments and actions. Furthermore, NTDs receive less attention than other medical issues^1 67^ such as HIV/AIDS, malaria and tuberculosis.^68^ Ng’etich *et al* found that HPs in Kenya were only prepared to participate in surveillance efforts for diseases that they deemed to be of priority such as immunisation.^69^ Because many NTDs, like schistosomiasis, are associated with morbidity instead of mortality, they may be considered as a lower priority.^68 70^ This is particularly true in situations where people are living in circumstances of high vulnerability due to lack of basic needs. Future interventions must include culturally tailored interventions and educational campaigns to enhance community engagement and promote the value of PC. By focusing on educational campaigns, we can address low health literacy by offering easily available information that align with community health attitudes and expectations, hence improving the acceptability of the supply side under the Levesque framework. This can also foster trust to healthcare professionals ensuring that schistosomiasis and other NTDs are considered as a priority for preventive healthcare actions. On the demand side, these interventions empower individuals with the knowledge to perceive risks and seek care, supported by caregivers who provide information and encouragement.

### Innovative strategies for MDA

As described in the results discussed above, this research highlights the necessity for innovative strategies to reach mobile populations. Adaptable MDA campaigns are essential to enhance access for these groups. Customising community engagement to the characteristics of Mali’s diverse mobile populations is vital. Given the dynamic nature of mobile populations, flexible mini-campaigns are recommended to effectively reach these populations. Collyer *et al* found that integrating a catch-up campaign in the first year of MDA for schistosomiasis, rapidly reduced prevalence and heavy-intensity infections.^71^ Mancarella *et al* demonstrated that the COVID-19 vaccine was effective using catch-up strategies in Italy.^72^ A similar study reported that catch-up campaigns have been successful in increasing routine vaccine coverage in Malawi.^73^ Innovative operational and implementation research can help to understand how to implement flexible interventions based on population-specific characteristics.

## Limitations

The study involved a purposive sample of mobile populations who claimed no prior treatment during MDA. This sample does not represent all mobile communities in Mali, thus limiting the generalisability of the study’s findings. It is possible that never-treated individuals may struggle to recall past experiences and emotions related to their condition more frequently than other social groups.^74^ Respondents may not provide all their reasons for never treatment, or they may report only recent reasons. They may be ashamed to give certain reasons, so as not to appear unrealistic or uncompliant to the interviewers. Brown *et al* have shown that social reactions can induce shame, leading to feelings of rejection, embarrassment and isolation, along with fear of exposure and judgement.^75^ To mitigate this effect in our study, we instructed interviewers to avoid judging participants’ words, to be culturally sensitive and to do everything possible to put participants at ease. Furthermore, questions surrounding never treatment had been previously validated in earlier studies.^14^,^1635^

### Standardised questions allow for the collection of qualitative data collection and analysis approach

These results contribute to the existing research on never treatment and provide input into the development of qualitative research questions to understand never treatment within programmatic evaluation responses and operational research. Questions should be carefully crafted to capture key barriers and facilitators for never treatment. Findings can help design programmatic responses and initiatives that aim to increase the engagement of IDPs, as well as nomadic and migrating groups in Mali and in other similar settings. Some recommendations for questions within cross-sectional surveys, qualitative research (IDIs and FGDs) and programmatic evaluation include:

- Have you ever declined to participate in MDA for (specific disease) in the past? If yes, please explain the main reasons or factors that influenced your decision.
- What are the reasons why you are never treated during MDA for NTDs? (an open question followed by: of that list, what is the primary reason (cite one only)).
- What needs to change for you to agree to take treatment in the next MDA round offered to you?
- In your opinion, what contributes to people never taking treatment in your community?
- In your opinion, what will make it easier for everyone in your community to access MDA for schistosomiasis? For onchocerciasis? For LF? For other PC-NTDs?
- What can be done to ensure that everyone participates in MDA targeting NTDs, including those who are never treated?
- What roles can health workers, other authorities and community members play in ensuring that everyone participates in MDA targeting NTDs?
- Do you participate in any other community-based health interventions? Why? Why not?

## Conclusion

This study highlights the challenges of never treatment in Mali’s schistosomiasis MDA programme among diverse mobile populations. The geographic mobility of populations poses a significant hurdle to the elimination of schistosomiasis as well as other PC-NTDs, demanding innovative strategies, such as flexible treatment methods (eg, mini-campaigns, specific CDD teams to target and treat mobile populations in their settlements, tailored educational campaigns and awareness, cross-sectoral collaboration) informed by subgroup-specific nuances. Levesque *et al*’s framework helps to conceptualise the elements of accessibility and availability of NTD treatment. By applying the Levesque framework, the study reveals that addressing these barriers requires a multifaceted approach that addresses both the supply side and the demand side, for example, improving MDA programmes by including community leaders to strengthen cultural acceptability (acceptability and ability to seek), ensuring the delivery processes are suited to the population’s needs (availability and accommodation and ability to reach) and creating tailored communication strategies that promote awareness and engagement (appropriateness and ability to engage). The results highlight the need for more flexible and context-specific approaches to achieve equitable access to MDA, hence, contributing to the overall goal of disease elimination in Mali.

## Supplementary material

10.1136/bmjgh-2024-015671 online supplemental figure 1

10.1136/bmjgh-2024-015671 online supplemental figure 2

10.1136/bmjgh-2024-015671 online supplemental file 1

10.1136/bmjgh-2024-015671 online supplemental file 2

## Data Availability

Data are available upon reasonable request.
