# Supplementary material for: Understanding the barriers and facilitators related to never treatment during mass drug administration among mobile and migrant populations in Mali: a qualitative exploratory study
Source: BMJ Glob Health. 2024 Oct 9;9(10):e015671. doi: 10.1136/bmjgh-2024-015671 (PMC11474861; doi:10.1136/bmjgh-2024-015671)
Supplement: online supplemental figure 1 [file bmjgh-9-10-s001.pdf]

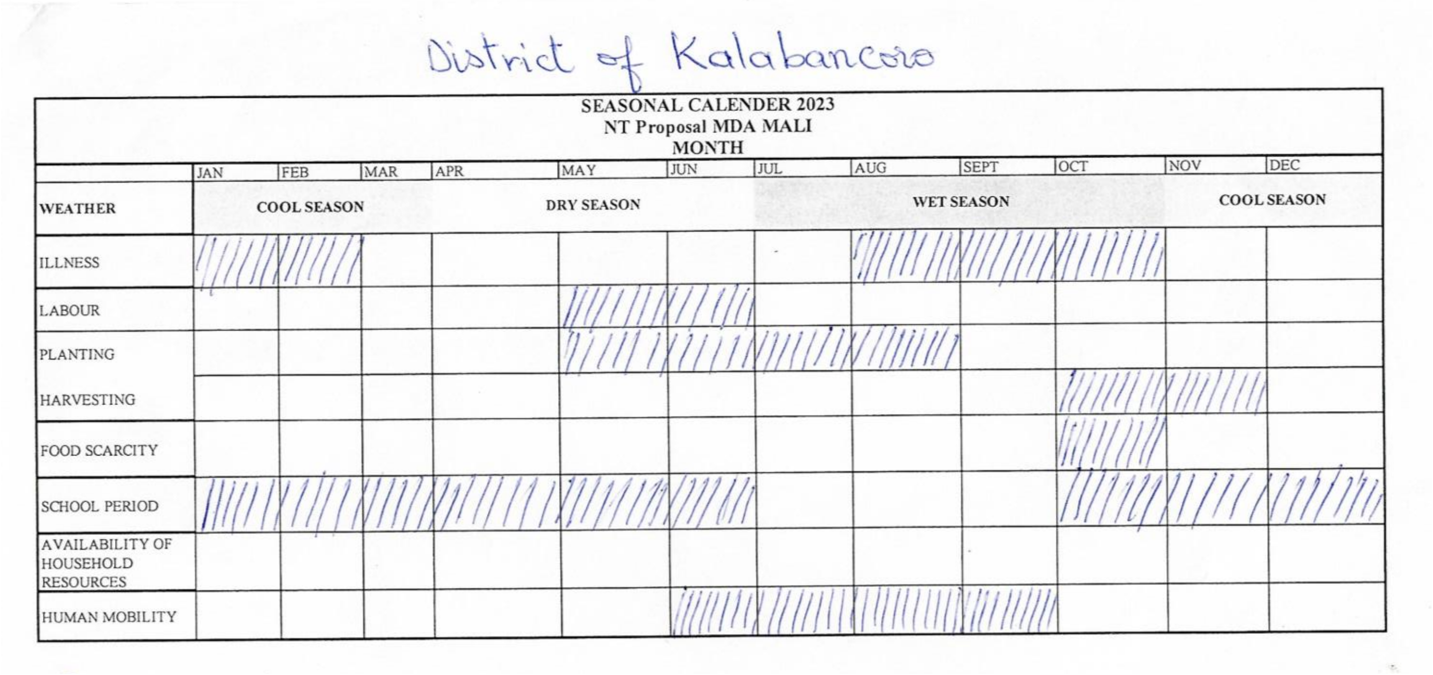

District of Kalabancoro

SEASONAL CALENDER 2023<br>NT Proposal MDA MALI<br>MONTH												
	JAN	FEB	MAR	APR	MAY	JUN	JUL	AUG	SEPT	OCT	NOV	DEC
WEATHER	COOL SEASON			DRY SEASON			WET SEASON				COOL SEASON	
ILLNESS	////	////						////	////	////		
LABOUR					////	////						
PLANTING					////	////	////	////				
HARVESTING										////	////	
FOOD SCARCITY										////		
SCHOOL PERIOD	////	////	////	////	////	////				////	////	////
AVAILABILITY OF HOUSEHOLD RESOURCES												
HUMAN MOBILITY						////	////	////	////			

Supplemental figure 1. Seasonal calendar district of Kalabancoro
