# Supplementary material for: Understanding the barriers and facilitators related to never treatment during mass drug administration among mobile and migrant populations in Mali: a qualitative exploratory study
Source: BMJ Glob Health. 2024 Oct 9;9(10):e015671. doi: 10.1136/bmjgh-2024-015671 (PMC11474861; doi:10.1136/bmjgh-2024-015671)
Supplement: online supplemental figure 2 [file bmjgh-9-10-s002.pdf]

District of Tominian

SEASONAL CALENDER 2023
NT Proposal MDA MALI
MONTH

	JAN	FEB	MAR	APR	MAY	JUN	JUL	AUG	SEPT	OCT	NOV	DEC
**WEATHER**	**COOL SEASON**			**DRY SEASON**			**WET SEASON**				**COOL SEASON**	
ILLNESS	///	///				///	///	///	///			
LABOUR					///	///						
PLANTING					///	///	///					
HARVESTING									///	///	///	
FOOD SCARCITY								///	///			
SCHOOL PERIOD	///	///	///	///	///	///				///	///	///
AVAILABILITY OF HOUSEHOLD RESOURCES												
HUMAN MOBILITY	///	///	///	///							///	///

Supplemental figure 2. Seasonal calendar district of Tominian
