# Supplementary material for: Understanding the barriers and facilitators related to never treatment during mass drug administration among mobile and migrant populations in Mali: a qualitative exploratory study
Source: BMJ Glob Health. 2024 Oct 9;9(10):e015671. doi: 10.1136/bmjgh-2024-015671 (PMC11474861; doi:10.1136/bmjgh-2024-015671)
Supplement: online supplemental file 1 [file bmjgh-9-10-s003.pdf]

Reflexivity Statement

As the primary researcher, I recognize that the methodology of this study has been influenced by my experiences and background. I am a man, Malian citizen, medical doctor and a PhD candidate in population health in Canada, with a professional focus on healthcare access among underserved populations. My personal experiences growing up in a rural community in Mali, where healthcare resources were often limited, have influenced my interest in this topic.

MS, AFD, ID, SOT, DT, FT, and BS conducted all the in-depth interviews and focus group of discussion. MS, AFD, YIC, SD, MK, TBN and AK conceived and conceptualized this study. Most of the investigators held a background in medicine, social epidemiology, and public health. It was important to us to have a team made up of both men and women to consider socio-cultural aspects and language barriers. Additionally, the interviewers' background in public health allowed them to develop a codebook that was informed by their prior knowledge of organizations' cultures and people in vulnerable circumstances access to mass drug administration against neglected tropical diseases allowed to highlight themes and sub-themes. This benefited the data collection as well as analysis as it shaped the thematic scope of the research, complementing the themes of the conceptual framework.

Throughout the research process, we remained aware of the potential for our own experiences and assumptions to influence data collection and interpretation. To mitigate this, we employed member-checking and peer debriefing, ensuring that participant voices were accurately represented and not overshadowed by our own perspectives. Moreover, we maintained a reflexive journal during fieldwork to track and reflect on any emerging biases or preconceptions. We aimed to remain as transparent and open as possible about our influence on the research process.

Our study requires a careful examination of power dynamics between the researcher and participants. If the researcher is perceived as an outsider, participants may withhold information due to distrust or fear of judgment, limiting the researchers' access to crucial insights. Power imbalances may arise from differences in education, socio-economic status, or cultural backgrounds, further influencing the openness of participants. We acknowledge that these

dynamics could affect the authenticity of the data. Therefore, we set out to mitigate this effect by ensuring a non-judgmental environment for honest dialogue during IDIs and FGDs. We started each interview with general conversation, to allow the participant to become comfortable with the research team and answering questions.

We also recognize that our data might also be limited by information bias such as social desirability, recall and self-reporting bias in the responses of participants. We recognized these limitations, and we took appropriate measures such as validating the topic guides before data collection, ensuring the confidentiality and anonymity of participants, reviewing the known characteristics of the population, reviewing the duration of the interview, checking the recall period and providing memory aids. During the piloting and review, in cases where the content of the interview was unclear or ambiguous due to the use of terminology, the team met to discuss it and find a consensus.

The strengths of this study lie in its relevance to the challenges of people living in vulnerable circumstances especially as NTD programs approach elimination. The study was conducted in collaboration with the NTD program in Mali, thus, the recommendations from the study can be used directly by the NTD program.
