# Supplementary material for: Understanding the barriers and facilitators related to never treatment during mass drug administration among mobile and migrant populations in Mali: a qualitative exploratory study
Source: BMJ Glob Health. 2024 Oct 9;9(10):e015671. doi: 10.1136/bmjgh-2024-015671 (PMC11474861; doi:10.1136/bmjgh-2024-015671)
Supplement: online supplemental file 2 [file bmjgh-9-10-s004.pdf]

Manuscript: Understanding the barriers and facilitators related to never treatment during mass drug administration among mobile and migrant populations in Mali, a qualitative exploratory study.

Consolidated criteria for reporting qualitative studies (COREQ): 32-item checklist

Developed from:

Allison Tong, Peter Sainsbury, Jonathan Craig, Consolidated criteria for reporting qualitative research (COREQ): a 32-item checklist for interviews and focus groups, International Journal for Quality in Health Care, Volume 19, Issue 6, December 2007, Pages 349–357, https://doi.org/10.1093/intqhc/mzm042

No. Item	Guide questions/description	Reported on Page #
**Domain 1: Research team and reflexivity**		
Personal Characteristics		
**1.** Interviewer/facilitator	Which author/s conducted the interview or focus group?	Page 29
**2.** Credentials	What were the researcher's credentials? E.g. PhD, MD	Page 1
**3.** Occupation	What was their occupation at the time of the study?	Page 1 and 10
**4.** Gender	Was the researcher male or female?	Page 10 and 11
**5.** Experience and training	What experience or training did the researcher have?	Page 9
Relationship with participants		
**6.** Relationship established	Was a relationship established prior to study commencement?	Page 10
**7.** Participant knowledge of the interviewer	What did the participants know about the researcher? e.g. personal goals, reasons for doing the research	From page 10 to 11
**8.** Interviewer characteristics	What characteristics were reported about the interviewer/facilitator? e.g. Bias, assumptions, reasons and interests in the research topic	Page 1, 10 and 26
**Domain 2: study design**		
Theoretical framework		
**9.** Methodological orientation and Theory	What methodological orientation was stated to underpin the study? e.g. grounded theory, discourse analysis, ethnography, phenomenology, content analysis	Page 8, 9 and 10

Participant selection		
**10.** Sampling	How were participants selected? e.g. purposive, convenience, consecutive, snowball	Page 8
**11.** Method of approach	How were participants approached? e.g. face-to-face, telephone, mail, email	Page 8 and 10
**12.** Sample size	How many participants were in the study?	Page 11
**13.** Non-participation	How many people refused to participate or dropped out? Reasons?	Page 8
Setting		
**14.** Setting of data collection	Where was the data collected? e.g. home, clinic, workplace	Page 6
**15.** Presence of non-participants	Was anyone else present besides the participants and researchers?	Page 8
**16.** Description of sample	What are the important characteristics of the sample? e.g. demographic data, date	Page 8 and 9
Data collection		
**17.** Interview guide	Were questions, prompts, guides provided by the authors? Was it pilot tested?	Page 8 and 9
**18.** Repeat interviews	Were repeat interviews carried out? If yes, how many?	N/A
**19.** Audio/visual recording	Did the research use audio or visual recording to collect the data?	Yes, page 9
**20.** Field notes	Were field notes made during and/or after the interview or focus group?	Page 8
**21.** Duration	What was the duration of the interviews or focus group?	Page 8
**22.** Data saturation	Was data saturation discussed?	Page 9
**23.** Transcripts returned	Were transcripts returned to participants for comment and/or correction?	N/A

**Domain 3: analysis and findings**		
Data analysis		
**24.** Number of data coders	How many data coders coded the data?	Page 9 and 10
**25.** Description of the coding tree	Did authors provide a description of the coding tree?	Page 9 and 10
**26.** Derivation of themes	Were themes identified in advance or derived from the data?	Page 8 and 9
**27.** Software	What software, if applicable, was used to manage the data?	Page 9 and 10
**28.** Participant checking	Did participants provide feedback on the findings?	N/A
Reporting		
**29.** Quotations presented	Were participant quotations presented to illustrate the themes/findings? Was each quotation identified? e.g. participant number	Yes, see results section Page 11 to 20
**30.** Data and findings consistent	Was there consistency between the data presented and the findings?	Yes, there was. From Page 11 to 20
**31.** Clarity of major themes	Were major themes clearly presented in the findings?	Yes. they were. From 11 to 20
**32.** Clarity of minor themes	Is there a description of diverse cases or discussion of minor themes?	Discussion of major and minor themes from page 20 to 25
